# Electroacupuncture preconditioning reduces ROS generation with NOX4 down-regulation and ameliorates blood-brain barrier disruption after ischemic stroke

**DOI:** 10.1186/s12929-016-0249-0

**Published:** 2016-03-08

**Authors:** Yeon Suk Jung, Sae-Won Lee, Jung Hwa Park, Hyung Bum Seo, Byung Tae Choi, Hwa Kyoung Shin

**Affiliations:** Korean Medical Science Research Center for Healthy-Aging, Pusan National University, Yangsan, Gyeongnam 626-870 Republic of Korea; Department of Korean Medical Science, School of Korean Medicine, Pusan National University, Yangsan, Gyeongnam 626-870 Republic of Korea; Division of Meridian and Structural Medicine, School of Korean Medicine, Pusan National University, Yangsan, Gyeongnam 626-870 Republic of Korea

**Keywords:** Focal cerebral ischemia, NADPH oxidase type 4, Electroacupuncture, Reactive oxygen species, Tight junction

## Abstract

**Background:**

Electroacupuncture (EA) is a modern application based on combination of traditional manual acupuncture and electrotherapy that is frequently recommended as an adjuvant treatment for ischemic stroke. EA preconditioning can ameliorate blood-brain barrier (BBB) dysfunction and brain edema in ischemia-reperfusion injury; however, its mechanism remains unclear. This study investigated the preventive effects of EA preconditioning, particularly on BBB injury, followed by a transient middle cerebral artery occlusion (MCAO) model in mice.

**Results:**

Mice were treated with EA (20 min) at Baihui (GV20) and Dazhui (GV14) acupoints once a day for 3 days before ischemic injury. Infarct volume, neurological deficits, oxidative stress, Evans blue leakage and brain edema were evaluated at 24 h after ischemia-reperfusion injury. EA preconditioning significantly decreased infarct volume and improved neurological function even after ischemic injury. In addition, both Evans blue leakage and water content were significantly reduced in EA preconditioned mice. Whereas the expression of tight junction proteins, ZO-1 and claudin-5, were remarkably increased by EA preconditioning. Mice with EA preconditioning showed the reduction of astrocytic aquaporin 4, which is involved in BBB permeabilization. In addition, we found that EA preconditioning decreased reactive oxygen species (ROS) in brain tissues after ischemic injury. The expression of NADPH oxidase 4 (NOX4), not NOX2, was significantly suppressed in EA preconditioned mice.

**Conclusions:**

These results suggest that EA preconditioning improve neural function after ischemic injury through diminishing BBB disruption and brain edema. And, the reduction of ROS generation and NOX4 expression by EA preconditioning might be involved in BBB recovery. Therefore, EA may serve as a potential preventive strategy for patients at high risk of ischemic stroke.

## Background

Stroke, which is one of the most common pathologies, has high morbidity and a high rate of long-term disability in industrialized countries [27]. Electroacupuncture (EA), which is the modern application of traditional acupuncture combined with electrotherapy, has been the most well-known complementary and alternative clinical approach to treatment of ischemic stroke for more than 3000 years. Although the clinical effectiveness of EA on ischemic stroke has been widely affirmed, its mechanisms of action are not fully understood. In animal experiments, repeated EA treatments have been shown to accelerate restoration of cerebral tissue lesion and function during cerebral ischemia-reperfusion using microPET imaging [25]. In addition, EA has been reported to induce brain ischemic tolerance via regulation of oxidative stress [45], maintenance of the blood-brain barrier (BBB) integrity [5] and inhibition of apoptosis through cannabinoid receptors [36, 37], adenosine receptor [38] and opiod receptors [39]. Our previous study showed that EA pretreatment induced tolerance against cerebral ischemic injury via upregulation of neurotrophic factors such as BDNF and SDF-1α [17]. However, more evidence is needed to suggest specific therapeutic targets strongly correlated with EA preconditioning, and the mechanism of cerebral ischemia tolerance induced by EA preconditioning remains unclear.

Ischemic stroke involves complicated pathological processes associated with multiple biological systems, the combined action of which determines the outcome of the ischemic event [27]. It is well known that brain edema is caused by water accumulation in the brain because of BBB disruption after ischemic stroke [42]; however, current clinical therapeutic methods for its treatment remain unsatisfactory. Cerebral ischemia-induced excessive production of reactive oxygen species (ROS) has been shown to be the main mechanism contributing to BBB disruption [15] and NADPH oxidases (NOX) are the primary sources of ROS following ischemic stroke, playing central roles in post-stroke BBB disruption [13].

This study was conducted to investigate the role of EA preconditioning in BBB dysfunction and brain damage elicited by focal cerebral ischemia-reperfusion and to explore the potential mechanisms involved. To accomplish this, we used a mouse model of middle cerebral artery occlusion (MCAO) to assess whether EA preconditioning reduces infarct volume and neurological deficits and if so, to elucidate whether the preventive effects are associated with inhibition of BBB disruption and brain edema, as well as the underlying mechanisms.

## Methods

### General surgical preparation

Male mice (C57BL/6J, 20–25 g) were housed under diurnal lighting conditions and allowed food and tap water *ad libitum*. All animal procedures were conducted in accordance with the institutional guidelines for animal research, and were approved by the University Animal Care and Use Committee (Permit Number: PNU-2011-000420). Computer-generated randomization was conducted using SigmaPlot 11.2 (Systat Software Inc, San Jose, CA) to allocate organisms to a vehicle and an EA group. After acquiring a number by computer-generated randomization, C57BL/6J male mice were allocated in a blinded fashion. Anesthesia was achieved by face mask-delivered isoflurane (2 % induction and 1.5 % maintenance, in 80 % N_2_O and 20 % O_2_). The depth of anesthesia was checked by the absence of cardiovascular changes in response to tail pinch. Rectal temperature was maintained at 36.5 °C–37.5 °C using a Panlab thermostatically controlled heating mat (Harvard Apparatus, Holliston, MA).

### EA stimulation

**Fig. 1:**
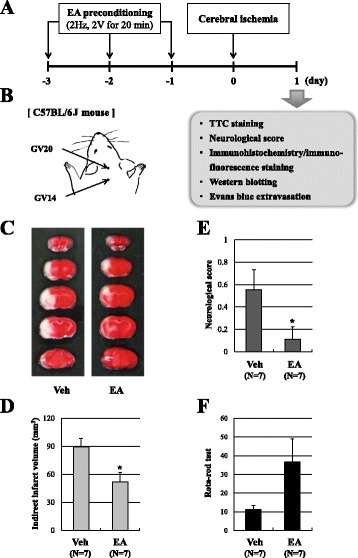
EA preconditioning reduced infarct size and improved neurological and motor function after focal cerebral ischemia. **a** A schematic diagram illustrating the chronological events of experiments. The mice received 20 min EA preconditioning once a day for three days prior to the ischemic event, after which focal cerebral ischemia was induced by middle cerebral artery occlusion (MCAO). **b** Mouse schematic showing the location of the acupuncture points used in the study. GV20 stands for ‘Baihui’, which is located at the right midpoint of the parietal bone. GV14 stands for ‘Dazhui’, which is located on the posterior midline and in the depression below the spinous process of the seventh cervical vertebra. **c** Representative photographs of coronal brain sections stained with 2,3,5-triphenyltetrazolium chloride (TTC) in vehicle (Veh) and EA-preconditioned mice. White region indicates the infarct area. **d**-**f** Quantification of the infarct volume (**d**), neurological deficit (**e**) and motor function (**f**) were assessed 24 h after MCAO in a blinded fashion. Data are expressed as the means ± SEM (*N* = 7). *P < 0.05 compared with the vehicle group (Veh)

Animals were anesthetized with isoflurane to avoid restraint stress. To assess the preconditioning effects of EA on ischemic brain injury, mice received EA preconditioning for 20 min once a day for three successive days before the ischemic event (Fig. 1a). The vehicle groups received only light isoflurane anesthesia for 20 min. The transpositional method, which locates the veterinary acupoints by transforming human acupoints onto animal anatomy, was used to determine the acupoints in mice [43]. The acupoint ‘Baihui (GV20)’, which is located at the right midpoint of the parietal bone, and ‘Dazhui (GV14)’, which is located on the posterior midline and in the depression below the spinous process of the seventh cervical vertebra, were stimulated as standard criteria Fig. 1b. To accomplish this, acupuncture needles (0.18 × 30 mm) were inserted into GV20 and GV14 to a depth of approximately 3 mm, after which they were stimulated at an intensity of 1 mA and a frequency of 2 Hz for 20 min [16, 23] using a Grass S88 electro stimulator (Grass Instrument Co., West Warwick, RI). The intensity was maintained just below the level that induced visible muscle contraction.

### Focal cerebral ischemia

Focal cerebral ischemia was induced by MCAO using a previously described intraluminal filament technique [10]. A fiber-optic probe was affixed to the skull over the MCA for measurement of the regional cerebral blood flow (CBF) using a PeriFlux Laser Doppler System 5000 (Perimed, Stockholm, Sweden). Baseline values were measured before internal carotid artery ligation (considered to be 100 % flow). MCA occlusion was induced by a silicon rubber-coated monofilament (Duccol Corporation, Redlands, CA) in the internal carotid artery, after which the monofilament was advanced to occlude the MCA. In all animals, the regional CBF was measured to confirm achievement of consistent and similar levels of ischemic induction. The filament was withdrawn 1 h after occlusion and reperfusion was confirmed using the laser Doppler. The surgical wound was sutured and mice were allowed to recover from anesthesia. The brains were removed 24 h after MCA occlusion. Cerebral infarct size was determined on 2,3,5-triphenyltetrazolium chloride (TTC)-stained, 2-mm-thick brain sections. Infarction areas were quantified using the iSolution full image analysis software (Image & Microscope Technology, Vancouver, Canada). To account for and eliminate the effects of swelling/edema, infarction volume was calculated via indirect measurement by summing the volumes of each section according to the following formula: contralateral hemisphere (mm^3^) - undamaged ipsilateral hemisphere (mm^3^) [24].

### Neurological score

Neurological deficit was scored in each mouse at 24 h after the ischemia reperfusion insult in a blinded fashion according to the following graded scoring system: 0 = no deficit; 1 = forelimb weakness and torso turning to the ipsilateral side when held by tail; 2 = circling to the affected side; 3 = unable to bear weight on the affected side; and 4 = no spontaneous locomotor activity or barrel rolling [22].

### Rota-rod test

Motor coordination and equilibrium were measured 24 h after cerebral ischemia using a rota-rod apparatus (Panlab S.L.U., Barcelona, Spain). After adaptation trials, each mouse was placed on the rotating rod for five trials at a speed of 18 rpm for 3 min and the time that an animal was able to hold itself on the rod was recorded.

### Detection of superoxide anion

ROS production in the brain was assessed using in vivo dihydroethidium (DHE, Life Technologies, Eugene, OR) staining. DHE, a cell-permeable oxidative-sensitive fluorescent dye, is oxidized to ethidium by superoxide, which, subsequently binds to DNA in the nucleus and emits red fluorescence. The frozen brains were cut at a thickness of 14 μm using a CM 3050 cryostat (Leica Microsystems), after which the sections were incubated with DHE (50 μM) in PBS for 10 min at 37 °C in a humidified chamber protected from light. The images of each section were captured with a Zeiss LSM 700 laser scanning confocal device (Carl Zeiss, Jena, Germany) and quantification of DHE-positive cells in three coronal sections in each animal was analyzed using the Image J program.

### Real-time PCR

Mice were deeply anesthetized with sodium thiopental 24 h after the induction of ischemia, after which they were perfused transcardially with cold PBS and the brain cortexes were collected. Total RNA was isolated from the ischemic cortex using TRIzol reagent^TM^ (Invitrogen, Carlsbad, CA) according to the manufacturer’s recommendations. The RNA was then reverse-transcribed for 1 h at 42 °C with Moloney Murine Leukemia Virus reverse transcriptase (Promega, Madison, WI) to produce cDNA. Real-time PCR was performed to quantify the amount of NOX2 and NOX4 mRNA in the ischemic brain using a Rotor-Gene Q real-time PCR system (Qiagen, Hilden, Germany) with SYBR Green PCR Master Mix (Qiagen), after which the results were normalized to GAPDH gene expression. All experiments were performed in triplicate and repeated at least three times. The following primer sequences were used:

- mouse Nox2 -557 to-725 (175 bp), 5′-CTGAAGGGGGCCTGTATGTG-3′ (forward)-, and 5′-ATGGCAAGGCCGATGAAGAA-3′ (reverse);
- mouse Nox4 -2478 to −5896 (119 bp), 5′-CCTCGCTGCAGTGTTCCTAA-3′ (forward)-, and 5′-GATTGGCTAAGGGGGAGCAG-3′ (reverse).

The threshold cycles (Ct) were used to quantify the mRNA expression of target genes.

### Western blotting

Proteins were subsequently isolated from the ischemic cortex according to the standard methods, separated by 12 % sodium dodecyl sulfate-polyacrylamide gel electrophoresis, and transferred onto a nitrocellulose membrane (Amersham Biosciences, Piscataway, NJ). Next, immunoblot analysis was performed with primary antibodies NOX2 (Abcam, Cambridge, UK), NOX4 antibody (Abcam), ZO-1 (Invitrogen Corporation, Carlsbad, CA), occludin (Invitrogen Corporation) and claudin-5 (Invitrogen Corporation) at 4 °C overnight, followed by horseradish peroxidase-conjugated anti-rabbit secondary antibody (Enzo, Farmingdale, NY) for 1 h. The intensity of chemiluminescence was measured using an ImageQuant LAS 4000 apparatus (GE Healthcare Life Sciences, Uppsala, Sweden). The membrane was then reprobed with anti-β-actin (Sigma-Aldrich) antibody as an internal control.

### Immunofluorescence staining

At 24 h after focal cerebral ischemia, mice were deeply anesthetized with sodium thiopental and subsequently perfused transcardially with cold PBS followed by 4 % paraformaldehyde for fixation. The brain of each mouse was then removed and further fixed in 4 % paraformaldehyde at 4 °C for 24 h, followed by cryoprotection in 30 % sucrose for 72 h at 4 °C. Next, the isolated brains were frozen in an optical cutting temperature medium for frozen tissue specimens (Sakura Finetek, Torrance, CA) and stored at −80 °C until examined. The frozen brains were cut at a thickness of 14 μm using a CM 3050 cryostat (Leica Microsystems, Wetzlar, Germany), after which the sections were immunostained with primary antibodies against GFAP (Dako, Glostrup, Denmark) and AQP4 (R&D Systems, Minneapolis, MN) at 4 °C. Following overnight incubation, the samples were incubated with FITC- or Texas red-conjugated secondary antibodies (Vector Laboratories, Inc., Burlingame, CA) for 2 h in the dark. The images of each section were captured with a Zeiss LSM 700 laser scanning confocal device (Carl Zeiss, Jena, Germany) and morphological analysis and quantification of positive cells was conducted using the Image J program. Positive cells were counted in random fields of view in the periinfarct area by an investigator blinded to the treatment groups. Counting was done in three adjacent brain sections. Three fields per predefined area of each brain section were counted for GFAP and AQP4 staining.

### Evans blue extravasation and water content

BBB integrity was evaluated by Evans blue extravasation. Briefly, Evans blue (2 % in saline, 4 ml/kg; Sigma-Aldrich) was administered intravenously at the onset of ischemia. Mice were deeply anesthetized with sodium thiopental, then transcardially perfused with PBS to remove the intravascular dye 24 h after cerebral ischemia. Next, each hemisphere was weighed, homogenized in 2 ml of *N*,*N*-dimethylformamide (Sigma-Aldrich), incubated for 24 h at 55 °C, and then centrifuged (13,000 rpm for 20 min). The absorbance of the supernatant at 620 nm was subsequently measured by spectrophotometry and the results were expressed as μg/g tissue calculated against a standard curve. The brain tissue water content was also measured by the wet and dry weight method 24 h after cerebral ischemia. To accomplish this, the hemispheres were weighed before and after drying at 100 °C for 48 h, and the percentage water content was calculated as 100 × (wet weight-dry weight)/wet weight.

### Data analysis

The data are expressed as the means ± the standard error of mean (SEM). The vehicle group was compared to the group treated with EA by unpaired t-tests. Differences were considered statistically significant, when the *P* values were < 0.05. All statistical analyses were performed using SigmaPlot 11.2 (Systat Software Inc).

## Results

### EA preconditioning attenuates brain damage after focal cerebral ischemia

To assess whether pretreatment with EA could attenuate brain damage following focal cerebral ischemia, the mice received 20 min EA preconditioning once a day for three days prior to the ischemia-reperfusion injury (Fig. 1a, b). TTC staining revealed that EA preconditioning significantly reduced infarct volume by 42 % from 89.4 ± 9.2 mm^3^ in the vehicle group to 51.9 ± 10.1 mm^3^ in the EA group following transient, 1 h MCA occlusion and 23 h reperfusion (*P* < 0.05; Fig. 1c, d). Concomitant with the infarct volume, we found that ischemia-induced neurological deficits were significantly improved in EA preconditioned mice 24 h after MCAO (Fig. 1e). In addition, the rota-rod test revealed that motor deficits tended to be recovered by EA preconditioning (Fig. 1f). These results showed that pretreatment with EA could improve tissue and functional outcome after ischemic brain injury.

### EA preconditioning prevents ischemia-induced blood-brain barrier destruction and brain edema

**Fig. 2:**
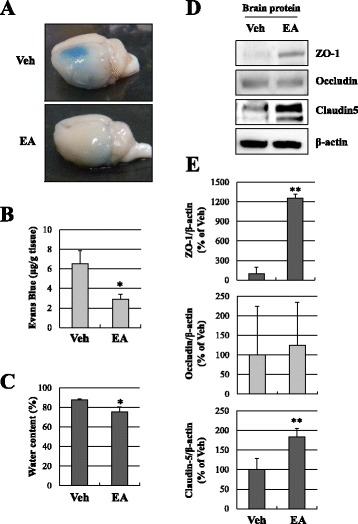
EA preconditioning increased tight junction proteins and attenuated Evans blue extravasation and brain edema. **a** Representative photographs of Evans blue leakage in vehicle or EA-preconditioned mice brains 24 h after focal cerebral ischemia. The blue area shows extravasated Evans blue, indicating BBB disruption. **b** Quantitative analysis of Evans blue leakage (*N* = 6, *P < 0.05 compared with the vehicle group). **c** Quantitative analysis of water content (*N* = 5, *P < 0.05 compared with the vehicle group). **d** Western blots of tight junction proteins, ZO-1, occludin and claudin-5, in the ischemic cortex 24 h after focal cerebral ischemia. β-Actin was used as an internal control. **e** Densitometric analysis of the western blot bands of ZO-1, occludin and claudin-5 (*N* = 4, ** *P* < 0.01 compared with the vehicle group). Data are expressed as the means ± SEM. Veh, vehicle group

To evaluate BBB permeability after ischemic brain injury, Evans blue extravasation was measured. EA preconditioning significantly reduced Evans blue extravasation in the ipsilateral hemisphere after focal cerebral ischemia (*P* < 0.05; Fig. 2a, b), suggesting that it alleviated the impairment of the BBB induced by cerebral ischemia. To examine EA preconditioning effects on post-ischemic edema formation, we evaluated brain water content at 24 h after reperfusion following MCAO. Concomitant with the results of Evans blue extravasation, the brain water content was significantly attenuated by EA preconditioning (13.9 % reduction of vehicle group, *P* < 0.05; Fig. 2c), suggesting that it inhibited edema formation following MCAO. To further investigate the mechanism of BBB disruption, we examined the expression of tight junction-related proteins ZO-1, occludin and claudin-5 in the ischemic cortex by Western blotting (Fig. 2d, e). Compared to the vehicle group, EA-pretreated mice displayed significantly increased expression of two tight junction proteins, ZO-1 and claudin-5 (Fig. 2d, e), suggesting that destruction of the BBB after focal cerebral ischemia was attenuated via increasing tight junction protein expression.

### EA preconditioning reduces astrocyte-AQP4 after ischemic brain injury

**Fig. 3:**
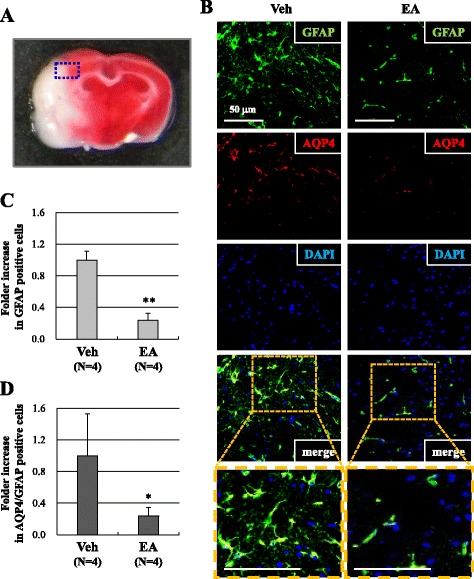
EA preconditioning reduced AQP4 expressing astrocytes after focal cerebral ischemia. **a** The blue rectangle illustrates the imaging field. **b** GFAP (green), AQP4 (red) and DAPI (blue) staining in the peri-infarct area of vehicle or EA-pretreated mouse brains 24 h after focal cerebral ischemia. Quantification was done in three adjacent brain sections and three fields per predefined area of each brain section were counted for GFAP and AQP4 staining. The co-expression of AQP4 and GFAP within the same cell is indicated by yellow (merge). **c**, **d** Quantification of GFAP(+) cells (**c**) or GFAP(+)/AQP4(+) cells (**d**) in the ischemic cortex. Data are expressed as the means ± SEM (*N* = 4). *P < 0.05, **P < 0.01 when compared with the vehicle group (Veh). The scale bar is 50 μm

We next determined whether EA preconditioning could attenuate astrocyte activation after ischemic brain damage by measuring the number of GFAP positive cells in the ischemic cortex using immunofluorescence (Fig. 3). The number of GFAP positive cells in the EA preconditioning group was significantly lower than that in the vehicle group (*P* < 0.01; Fig. 3b, c), indicating that EA might inhibit astrocytes in the ischemic cortex. Double immunofluorescence staining was then used to detect the expression and localization of aquaporin 4 (AQP4) in the brain. AQP4 is a water channel expressed in astrocyte end-feet lining the BBB [1]. AQP4 was highly expressed in brain of vehicle group and co-expressed in GFAP-positive cells. The GFAP(+)/AQP4(+) immunofluorescence was significantly reduced in the EA preconditioning group (*P* < 0.05; Fig. 3b, d). These results suggest that EA preconditioning could suppress AQP4 expressing astrocytes in ischemic brain.

### NOX4, not NOX2, is involved in EA preconditioning mediated ROS reduction

**Fig. 4:**
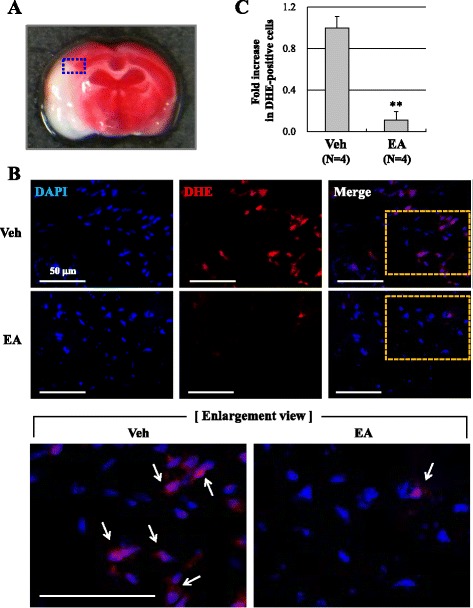
EA preconditioning reduced ROS production after focal cerebral ischemia. **a** The blue rectangle illustrates the imaging field. **b** Dihydroethidium (DHE, red, arrows) fluorescence images show the superoxide signal taken from the ischemic cortex 24 h after focal cerebral ischemia. The mice received EA (20 min) once a day for three days prior to the ischemic event. DAPI (blue), nucleus. Scale bar is 50 μm. **c** Quantification was done in three adjacent brain sections and three fields per predefined area of each brain section were counted for DHE staining in the ischemic cortex. Data are expressed as the means ± SEM (*N* = 4). ** *P* < 0.01 when compared with the vehicle group (Veh)

**Fig. 5:**
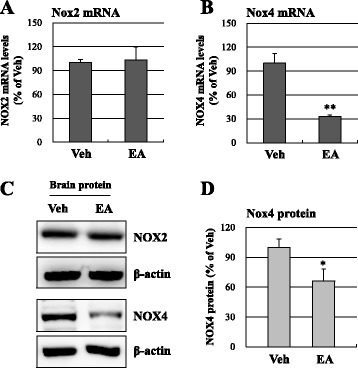
EA preconditioning down-regulated the expression of NOX4, not NOX2, after focal cerebral ischemia. **a**, **b** Real-time PCR shows the mRNA levels of NOX2 and NOX4 in the ischemic brain tissues 24 h after focal cerebral ischemia. (*N* = 4, ** *P* < 0.01 compared with the vehicle group). **c** Western blots of NOX2 and NOX4 in the ischemic cortex 24 h after focal cerebral ischemia. β-Actin was used as an internal control. **d** Densitometric quantification graph of the western blot bands of NOX4. Data are expressed as the means ± SEM (*N* = 4). *P < 0.05 when compared with the vehicle group (Veh)

Excessive production of ROS following cerebral ischemia has been regard as one of the main causes of BBB disruption [15]. We observed the ROS production with DHE, a marker for superoxide (Fig. 4). The intensity of red fluorescence of DHE-positive cells was markedly reduced in the ischemic cortex of the EA preconditioning group relative to the vehicle group (0.11 ± 0.08-fold, *P* < 0.01; Fig. 4b, c). We next examined the two most important NOX isoforms of superoxide formation, NOX2 and NOX4, by real-time PCR and western blotting (Fig. 5). Only NOX4 mRNA expression (32.6 ± 2.3 % of the vehicle group, *P* < 0.01; Fig. 3b), but not NOX2 mRNA (Fig. 5a, b), was decreased by EA preconditioning in the ischemic cortex. We confirmed NOX protein levels in the ischemic cortex at 24 h after MCAO. Correspondingly, the protein level of NOX4 was significantly suppressed in EA preconditioning group (66.2 ± 12.0 % of vehicle group, *P* < 0.05; Fig. 5c, d). Taken together, these results suggested that EA preconditioning is a potent suppressor of ROS production after ischemic injury and NOX4 might have a role in EA preconditioning mediated ROS reduction.

## Discussion

**Fig. 6:**
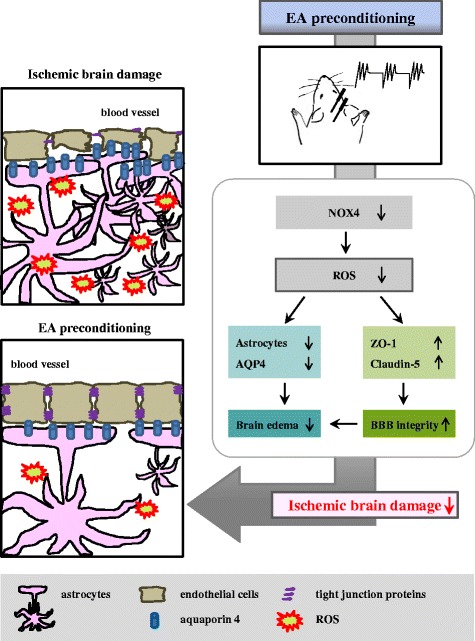
Schematic representation of the importance of EA preconditioning for attenuating cerebral ischemia-reperfusion injury

Our study showed that EA preconditioning for three days prior to ischemia-reperfusion injury significantly reduced infarct size and improved neurological function after focal cerebral ischemia. Staining of DHE showed that EA preconditioning markedly inhibited superoxide production in the ischemic cortex, which was accompanied by a reduction of NOX4 expression. Moreover, the results of immunofluorescence staining showed a significant decrease in the expression of GFAP(+)/AQP4(+) cells after cerebral ischemia following EA preconditioning. Regarding the BBB permeability, less Evans blue extravasation and water contents were observed in the EA preconditioning group. In addition, expression of ZO-1 and claudin-5 was increased in the ischemic cortex in response to EA. Our findings indicated that EA preconditioning could attenuate cerebral ischemia-reperfusion injury, and the underlying mechanism was related to reciprocal regulation; down-regulation of NOX4 and ROS production, while up-regulation of tight junction proteins (Fig. 6).

Pre-conditioning is as a potent endogenous protective response that activates several endogenous signaling pathways, resulting in tolerance against ischemia [4]. EA pretreatment has been shown to induce ischemic tolerance mimicking ischemic pretreatment via enhancing antioxidant activity [45], regulation of the endocannabinoid system [36, 37] and neurotrophic factors [17] and involvement of adenosine receptor [38] and opiod receptors [39], suggesting that EA pretreatment may be a promising preventive strategy for patients with high risk of acute ischemia brain injury. Since EA is economical, easily performed, and has low side effects, it is clinically applicable for prevention, and not just treatment of ischemic stroke. This study demonstrated that, similar to the ischemic tolerance induced by ischemic preconditioning, 20 min of EA treatment once a day for three days prior to the ischemia-reperfusion injury significantly decreased infarct volume and improved neurological function after focal cerebral ischemia in mice (Fig. 1). Thus, the results from both functional and tissue data suggest that EA pretreatment could induce tolerance to cerebral ischemic insult.

Although EA pretreatment could have a preventive effect against cerebral ischemic injury, there are several characteristics of EA treatment that differ from other treatment methods, especially the acupoint specificity. Previous studies have shown that EA stimuli at the Baihui acupoint (GV20) efficiently reduced brain infarction and neurological symptoms [5, 37]. In our study, the Baihui (GV20) and Dazhui (GV14) acupoints were chosen because the theory of meridians in traditional Korean medicine indicates that they are closely related to the brain and spinal cord and they were commonly used to treat stroke in ancient Korea. Recently, there are several papers that EA stimuli at the Baihui and Dazhui acupoints have a protective effect on ischemic brain damage using different ischemia models (MCAO model and photothrombotic cortical infarction model) and different animals (rats and mice) [3, 17, 18]. In the present study, treatment of EA stimulation at GV20 and GV14 before focal cerebral ischemia improves tissue and functional recovery. However, this finding raises the concern that the recovery effects were not mediated by acupuncture, but by electrical stimulation. As a negative control, we investigated the mice received the same electrical stimulation at non-acupuncture points in our previous publication [16]. We observed that the mice received the same electrical stimulation at non-acupuncture points (lateral points to aforementioned acupoint by approximately 1 mm apart) did not show any protective responses [16]. This result suggested that the effects of EA at GV20 and GV14 cannot be attributed to electrical stimulation alone.

Several researchers have demonstrated that the blockade of free radicals and vasogenic edema may be responsible for the therapeutic action of EA [33, 34, 44]. Induced excessive production of ROS represents the major mechanism contributing BBB disruption following ischemic stroke [39]. In addition, NOXs are the major sources of ROS that lead to BBB disruption following cerebral ischemia [13]. It is well known that there are three types of NOXs expressed in the central nerve system: NOX1, NOX2, and NOX4 [12]. Of these, NOX2 and NOX4 are upregulated within 24 h, after which subsequent ROS generation contributes to BBB disruption, inflammation, and post-ischemic neuronal injury [8, 21]. In addition, NOX4 KO mice showed profoundly reduced oxidative stress, BBB dysfunction, and neuronal apoptosis 24 h after transient MCAO, whereas deficiency of NOX1 or NOX2 had no impact on infarct size or functional outcome [21]. Therefore, preventing the generation of ROS early in the process by blocking NOX4 might be a potential therapeutic strategy for treatment of ischemic stroke.

Previous studies indicated that EA increased the activation of anti-oxidant enzymes [33, 34] and inhibited oxidative stress in ischemic injury [2]. Thus, our study suggests that the preventive effects of EA on brain ischemic injury involve its ability to attenuate BBB disruption and brain edema (Fig. 2), and that this BBB protection may involve the suppression of NOX4 expression and ROS production (Figs. 4, 5). Our results were supported by a recent report that EA pretreatment inhibits NOX-mediated oxidative stress in diabetic mice with cerebral ischemia [9]. Therefore, we suggest that decreasing ROS generation with NOX4 expression is the underlying mechanism of EA protection following focal cerebral ischemia. Because EA remarkably decreased NOX4 expression (Fig. 5), it might be interesting how EA regulate NOX4 expression. We searched for transcription factor binding sites in the NOX4 gene promoter using the bioinformatics site (www.genecards.org). We speculated that the transcription factors for NOX4 expression are suppressed by EA. The binding sites for ATF-2, AP-1, c-Jun, Nkx2.5 and p53 exist in NOX4 promoter region (www.genecards.org). Interestingly, EA down-regulates the ATF-2 protein expression in a rat model of inflammatory pain and reduce the inflammatory pain [7]. In the inflammatory rat model, EA decreases p-c-Jun protein level and the DNA binding activity of AP-1 [6]. In addition, EA exerts anti-apoptotic effects in rat cerebral ischemia model, and EA protects against pyramidal cell death by blocking p53 expression in vascular dementia rats [19, 46]. The mechanism involvement in the down-regulation of NOX4 by EA is less clear. However, we believe that the EA-mediated suppression of regulatory factors such as ATF-2, AP-1, c-Jun and p53 may be among the contributing factors. Further research is needed to clarify this issue.

AQP4 is the most abundant water channel in brain tissue, especially in perivascular end-feet of astrocytes lining the BBB and brain-cerebrospinal fluid interfaces [28]. An increase in AQP4 expression may aggravate injury to endothelial cells and tight junctions, promote BBB destruction, and lead to water entering brain tissues, resulting in an increase in brain water content and the formation of brain edema. Cerebral edema is a major and potential fatal complication of acute ischemic stroke. There is accumulating evidence that cerebral ischemia upregulates AQP4 expression, increases BBB permeability, and induces brain edema, which exacerbates ischemic brain injury [30]. AQP4 immunoreactivity was found to be increased with brain edema formation in human autopsy [1], and AQP4 knockout mice ameliorate brain swelling and improve outcome following ischemia [14, 26], while an accelerated progression of cytotoxic brain swelling was observed in mice overexpressing glial cell AQP4 [41]. In addition, the AQP4 inhibitor, TGN-020, ameliorated cerebral edema in ischemic mouse brain [11], while mesenchymal stem cells maintained BBB integrity by inhibiting AQP4 upregulation after cerebral ischemia [35]. Thus, regulation of AQP4 after cerebral ischemia might provide a therapeutic option for reducing brain edema. EA was recently reported to significantly attenuate expression of the water channel proteins, AQP4 and AQP9, in the ischemic brain [40]. In addition, EA markedly reduced neurological deficits, decreased corpus striatum AQP4 protein and mRNA expression, and relieved damage to the BBB in a rat model of cerebral ischemia-reperfusion injury [31]. We observed that the numbers of GFAP(+)/AQP4(+) cells were significantly lower at the cortical border zone in the EA preconditioning group (Fig. 3). This finding also indicated that the neuroprotective mechanism of EA preconditioning could be related to the down-regulation of AQP4(+) astrocytes.

After ischemic brain injury, the BBB integrity was disrupted, which was followed by an increase in vascular permeability and brain edema and secondary brain injury, exacerbating the initial ischemic injury [27]. Therefore, maintenance of BBB integrity has been one of major targets for protecting the brain from ischemic stroke. Tight junction proteins are the core portions of the BBB, which is located in the tightly sealed monolayer of brain endothelial cells [29]. Tight junction proteins such as ZO-1, occludin and claudin-5 have been shown to be reduced in experimental neurological disease models, which consequently compromised the integrity of the BBB [20]. In addition, experimental evidence and clinical data suggest that ROS disrupt the BBB by degrading the tight junctions [32]. EA pretreatment for five days was recently shown to significantly reduce BBB permeability and brain edema, which were correlated with alleviation of the degradation of tight junction proteins occludin and claudin-5 following cerebral ischemia-reperfusion in rats [47]. We here demonstrated that EA pretreatment increased the expression of tight junction protein such as ZO-1 and claudin-5, which corresponded to its protective effect against BBB disruption and edema in cerebral ischemic brains (Fig. 2). These findings are in line with those of previous studies that showed EA can alleviate cerebral edema of rats after ischemia using diffusion-weighted MRI [44]. Dong *et al*. also observed that EA preconditioning protected BBB integrity and brain edema via reduction of MMP-9 expression and activity [5]. Therefore, BBB destruction and brain edema after ischemic brain injury could be effectively rescued by EA pretreatment, at least a result of increased expression of ZO-1 and claudin-5.

## Conclusions

Our current study revealed the effectiveness and mechanism of EA preconditioning on the reduction of infarct volume and neurological deficits after ischemia-reperfusion injury. We demonstrated that EA preconditioning can delay or ameliorate the development of ischemic brain edema, and that this may be achieved via down-regulation of ROS generation, NOX4 expression and AQP4(+) astrocytes. Moreover, EA preconditioning up-regulates tight junction proteins, resulting in cerebral ischemia tolerance. Therefore, decreasing BBB disruption and brain edema via regulating NOX4 expression and ROS production by EA preconditioning may be an effective preventive strategy for reducing further ischemic brain damage.
